# Morphologic brain network predicts levodopa responsiveness in Parkinson disease

**DOI:** 10.3389/fnagi.2022.990913

**Published:** 2023-01-05

**Authors:** Yongsheng Xie, Chunyan Gao, Bin Wu, Liling Peng, Jianjun Wu, Liqin Lang

**Affiliations:** ^1^Department of Neurosurgery, Huashan Hospital, Fudan University, Shanghai, China; ^2^National Center for Neurological Disorders, Shanghai, China; ^3^Shanghai Key Laboratory of Brain Function Restoration and Neural Regeneration, Shanghai, China; ^4^State Key Laboratory of Medical Neurobiology and MOE Frontiers Center for Brain Science, School of Basic Medical Sciences and Institutes of Brain Science, Fudan University, Shanghai, China; ^5^Department of Nursing, Huashan Hospital, Fudan University, Shanghai, China; ^6^Department of Neurology and National Clinical Research Center for Aging and Medicine, Huashan Hospital, Fudan University, Shanghai, China; ^7^Shanghai Universal Medical Imaging Diagnostic Center, Shanghai, China

**Keywords:** Parkinson disease, deep brain stimulation, Levodopa challenge test, morphologic brain network, connectome

## Abstract

**Background:**

The levodopa challenge test (LCT) has been routinely used in Parkinson disease (PD) evaluation and predicts the outcome of deep brain stimulation (DBS). Guidelines recommend that patients with an improvement in Unified Parkinson’s Disease Rating Scale (UPDRS)-III score > 33% in the LCT receive DBS treatment. However, LCT results are affected by many factors, and only provide information on the immediate effectiveness of dopamine. The aim of the present study was to investigate the relationship between LCT outcome and brain imaging features of PD patients to determine whether the latter can be used to identify candidates for DBS.

**Methods:**

A total of 38 PD patients were enrolled in the study. Based on improvement in UPDRS-III score in the LCT, patients were divided into low improvement (PD-LCT-L) and high improvement (PD-LCT-H) groups. Each patient’s neural network was reconstructed based on T1-weighted magnetic resonance imaging data using the Jensen–Shannon divergence similarity estimation method. The network was established with the multiple kernel support vector machine technique. We analyzed differences in individual morphologic brain networks and their global and local metrics to determine whether there were differences in the connectomes of PD-LCT-L and PD-LCT-H groups.

**Results:**

The 2 groups were similar in terms of demographic and clinical characteristics. Mean ± SD levodopa responsiveness was 26.52% ± 3.47% in the PD-LCT-L group (N = 13) and 58.66% ± 4.09% in the PD-LCT-H group (*N* = 25). There were no significant differences between groups in global and local metrics. There were 43 consensus connections that were affected in both groups; in PD-LCT-L patients, most of these connections were decreased whereas those related to the dorsolateral superior frontal gyrus and left cuneus were significantly increased.

**Conclusion:**

Morphologic brain network assessment is a valuable method for predicting levodopa responsiveness in PD patients, which can facilitate the selection of candidates for DBS.

## Introduction

Parkinson disease (PD) is the second most common neurodegenerative motor disorder and affects more than 6 million people worldwide (Feigin et al., 2019). It is characterized by the degeneration of dopaminergic neurons and pathologic formation of Louis corpuscles, leading to motor symptoms such as tremor, muscle stunting, movement retardation, and posture imbalance as well as non-motor manifestations such as sleep, olfactory, cognitive, and mental disorders and autonomic dysfunction (Poewe et al., 2017). According to epidemiologic studies conducted in Europe and the United States, the prevalence rate of PD is 1% in people over the age of 60 years and > 4% in people over the age of 80 years, with the rates expected to rise over the next few decades (Balestrino and Schapira, 2020). The diagnosis of PD is mainly made and the severity determined through clinical examination and follow-up (Tolosa et al., 2021). In China, the treatment approach for PD is long-term, multidisciplinary integrated therapy (Chen S. et al., 2016).

Deep brain stimulation (DBS), especially closed-loop or adaptive DBS, is an essential aspect of PD treatment (Habets et al., 2018). Patients undergo brain imaging including magnetic resonance imaging (MRI) and positron emission tomography–computed tomography (PET-CT) before DBS is performed. The levodopa challenge test (LCT) is also widely recommended before the procedure (Saranza and Lang, 2021). A 30% or 33% improvement in Unified Parkinson’s Disease Rating Scale (UPDRS)-III score in the LCT has been set as a threshold for selecting candidates for DBS (Defer et al., 1999); however, it is unclear how this can predict the effectiveness of the DBS operation. It was reported that patients with a motor symptom improvement rate of <30% in the l-dopa impact test responded well to DBS (Zheng et al., 2021); and at our center, DBS was effective in some patients with <33% improvement in the LCT. As the LCT does not fully reflect brain function, it is important to establish other methods for predicting the response of PD patients to DBS.

Neuroimaging studies have revealed structural and functional alterations in multiple brain networks in PD (Ji et al., 2018). For example, changes in the brain network observed by ^18^F-fluorodeoxyglucose (FDG)-PET/CT can be used for PD diagnosis and treatment selection for patients (Li et al., 2021a) Voxel-based morphometry (VBM) is a relatively new approach for analyzing MRI data that has objective and quantitative advantages (Lenka et al., 2015). The aim of this study was to determine whether morphologic brain network changes observed by MRI and VBM in patients with PD are associated with LCT results, and can thus be used to identify patients who are likely to respond well to DBS.

## Materials and methods

### Study population

A total of 38 patients diagnosed with idiopathic PD according to International Parkinson and Movement Disorder Society diagnostic criteria were retrospectively enrolled in the study. All patients underwent implantation surgery for DBS at Huashan Hospital, Fudan University from January 2020 to December 2021. Patients with a history of head trauma, stroke, intracranial tumor, hydrocephalus, and psychiatric illness were excluded. Medical records were thoroughly reviewed to collect detailed information. Written, informed consent was provided by each patient or their legal guardians. The study was approved by the Institutional Review Board of Huashan Hospital and Medical Ethics Committee of Huashan Hospital, Fudan University, Shanghai, China. The procedures used in this study adhered to the tenets of the Declaration of Helsinki.

### LCT

The LCT was administered to patients by experienced neurologists at Huashan Hospital. To induce the “off” medication state, dopamine receptor agonists were stopped 72 h before the test, followed by levedopa and other dopaminergic medications 12 h before the test. After the first evaluation of UPDRS-III score as the “off” baseline, 10 mg domperidone was administered orally, followed by a dose of 150% of the standard first morning levodopa equivalent dose 30 min later. UPDRS-III score was assessed every 30 min until 4 h after levodopa intake. The lowest score was recorded as the peak “on” value. Levodopa responsiveness (LR) was calculated as follows: % LR = (“off” UPDRS-III score − peak “on” UPDRS-III score) / “off” UPDRS-III score × 100%. During the test, patients’ heart rate and blood pressure were monitored and any adverse events were recorded.

### Image acquisition and preprocessing

MRI was performed on an 3 T Ingenia scanner (Koninklijke Philips N.V., Amsterdam, The Netherlands). Structural 3D T1-weighted images were acquired with the following parameters: axial section thickness, 1.0 mm; no gap; repetition time, 6,900 ms; echo time, 2.9 ms; field of view, 240 × 224 mm × 170 mm; matrix size, 240 × 240 × 170; voxel size, 1 × 1 × 1 mm^3^; and signal-to-noise ratio (SNR), 1.004. Imaging data were preprocessed using the Computational Anatomy Toolbox (CAT12; http://www.neuro.uni-jena.de/cat/) from Statistical Parametric Mapping 12 (SPM12; http://www.fil.ion.ucl.ac.uk/spm/software/spm12/). Gray matter (GM) was segmented with default parameters and spatially normalized to the Montreal Neurological Institute space, followed by nonlinear modulation to compensate for potential bias. After these steps, a GM volume map was obtained for each subject (a voxel size of 1.5 × 1.5 × 1.5 mm). Spatial smoothing (Gaussian kernel with 6-mm full width at half maximum) was further applied to enhance the SNR of the GM volume map of each patient (voxel size of 1.5 × 1.5 × 1.5 mm). The cerebral cortex was divided into 90 regions (45 per cerebellar hemisphere) based on automatic anatomical labeling (AAL) (Tzourio-Mazoyer et al., 2002).

### Individual Jensen–Shannon divergence similarity estimation morphologic brain network construction

Early-stage PD with cognitive impairment can be predicted based on topologically convergent and divergent GM networks (Suo et al., 2021a). The distribution divergence-based method has been used in morphologic brain network investigations including in PD (Yoo et al., 2017; Fiorenzato et al., 2019; Yang et al., 2021). Specifically, Kullback–Leibler (KL) divergence (Suo et al., 2021b) was applied to construct the network according to the following formula:


(1) $$D_{KL}\;\left(\mathbb{P}∥\mathbb{Q}\right)=\underset{X}{\int }\left(\mathbb{P}\left(x\right)\log\frac{\mathbb{P}\left(x\right)}{\mathbb{Q}\left(x\right)}+\mathbb{Q}\left(x\right)\log\frac{\mathbb{Q}\left(x\right)}{\mathbb{P}\left(x\right)}\right)dx$$

where P and Q represent probability density functions (PDFs) of voxel intensities from a pair of regions of interest (ROIs). We applied the JSSE to construct individual mathematical relationships for any 2 ROIs to achieve a more accurate and symmetric estimate of morphologic brain connectivity. Based on the ROI parcellation from the AAL atlas, a 90 × 90 region correlation matrix was generated for each patient and the intensity of the ROI was extracted to estimate the corresponding PDF. Morphologic connections were derived as the JS divergence (relative entropy) using the following equation:


(2) $$D_{JS}\;\left(\mathbb{P}∥\mathbb{Q}\right)=\frac{1}{2}\left[D_{KL}\left(\mathbb{P}∥M\right)+D_{KL}\left(\mathbb{Q}∥M\right)\right]\;$$

where $D_{KL}$ are the KL divergence. The JS divergence was used as a measure of morphologic connectivity to generate the adjacency matrix.

### Computation of graph metrics

Global and local graph metrics of the morphologic brain network were determined by graph theoretical network analysis (Bullmore and Sporns, 2009) to evaluate individual connectivity patterns. Global graph metrics included clustering coefficient (Cp), small world (σ), global efficiency (E_global_), local efficiency (E_local_), characteristic path length (Lp), normalized clustering coefficient (γ), normalized characteristic path length (λ), and modularity score (Termenon et al., 2016) and also assortativity, and nodal graph metrics included degree centrality (DC), nodal efficiency (Ne), betweenness centrality (BC), shortest path length, and nodal clustering coefficient (Xu et al., 2020).In this study, synchronization had been adopt. Before calculating the sum of the corresponding node attribute values under the sparse threshold, we compared the network size of different sparse thresholds (0.02–0.5 in steps of 0.01). We then applied the sum of the values of each node as an attribute to train the classifier so that only one value corresponded to a graphical measure.

### Feature combination

In order to more accurately predict levodopa responsiveness, connection weight, global metrics, and nodal metrics were combined with the multikernel support vector machine (MK-SVM) technique (Xu et al., 2021). In a case with *n* training samples with connection values and graph metrics,$\;x_{i}^{1}$, $x_{i}^{2}$, and $x_{i}^{31}$ represented the connection weight, global metrics, and nodal metrics, respectively, of the *i*^th^ sample. With $y_{i}\in \left\{1,-1\right\}\;$as the corresponding label, the following problem was solved:


(3) $$\begin{array}{l} \underset{W}{\min}\frac{1}{2}\overset{3}{\underset{m=1}{\sum }}\beta_{m}w^{m2}+C\overset{n}{\underset{i=1}{\sum }}\xi_{i}\\ s.t.y_{i}\left(\overset{2}{\underset{m=1}{\sum }}\beta_{m}{\left(w^{m}\right)}^{T}\phi^{m}\left(x_{i}^{m}\right)+b\right)\geq 1-\xi_{i}\\ \xi_{i}\geq 0,i=1,2,\ldots ,n \end{array}$$

where $\phi^{m}$ represents the transform from the original space in *m*^th^ data to the Represent Hilbert Kernel Space (RHKS), $w^{m}$ is the hyperplane in RHKS, and $\beta^{m}$ is the corresponding combined weight of the *m*^t^h attribute. The dual form of MK-SVM is represented as follows:


(4) $$\begin{array}{l} \underset{\alpha }{\max}\overset{n}{\underset{i=1}{\sum }}\alpha_{i}-\frac{1}{2}\underset{i,j}{\sum }\alpha_{i}\alpha_{j}y_{i}y_{j}\overset{3}{\underset{m=1}{\sum }}\beta_{m}k^{m}\left(x_{i}^{m},x_{j}^{m}\right)\;\\ s.t.\overset{n}{\underset{i=1}{\sum }}\alpha_{i}y_{i}=0\\ 0\leq \alpha_{i}\leq C,i=1,2,\ldots n \end{array}$$

where $k\left(x_{i}^{m},x_{j}^{m}\right)=\phi^{m}{\left(x_{i}^{m}\right)}^{T}\phi^{m}\left(x_{j}^{m}\right)$ is the kernel matrix of the *m*th data. After training the model, we tested the new sample: *x*$=\left\{x^{1},x^{2},\cdots ,x^{M}\right\}$. The kernel between the new test sample and *i*th training sample in the *m*th modality was defined as $k\left(x_{i}^{m},x^{m}\right)=\phi^{m}{\left(x_{i}^{m}\right)}^{T}\phi^{m}\left(x^{m}\right)$.

The predictive level based on MK-SVM was formulated using the following equation.


(5) $$f\left(x_{1},x_{2},\ldots ,x_{M}\right)=sign\left(\overset{n}{\underset{i=1}{\sum }}y_{i}\alpha_{i}\overset{M}{\underset{m=1}{\sum }}\beta_{m}k^{m}\left(x_{i}^{m},x^{m}\right)+b\right)$$

To evaluate the gain in predictive performance of the combined information (ie, connection and global and nodal graph metrics in addition to LCT results), we employed the most commonly used and simplest linear kernel $k\left(x_{i}^{m},x_{j}^{m}\right),$ according to the following equation.


(6) $$k^{m}\left(x_{i}^{m},x_{j}^{m}\right)=x_{i}^{m}{}^{T}x_{j}^{m}$$

### Feature selection and validation

To determine whether there were differences between the 2 groups, the strictest nested leave-one-out cross-validation (CV) (Li et al., 2017) was used in the construction of connections by combining the information from connection weights and global and nodal graph metrics.

**Figure 1:**
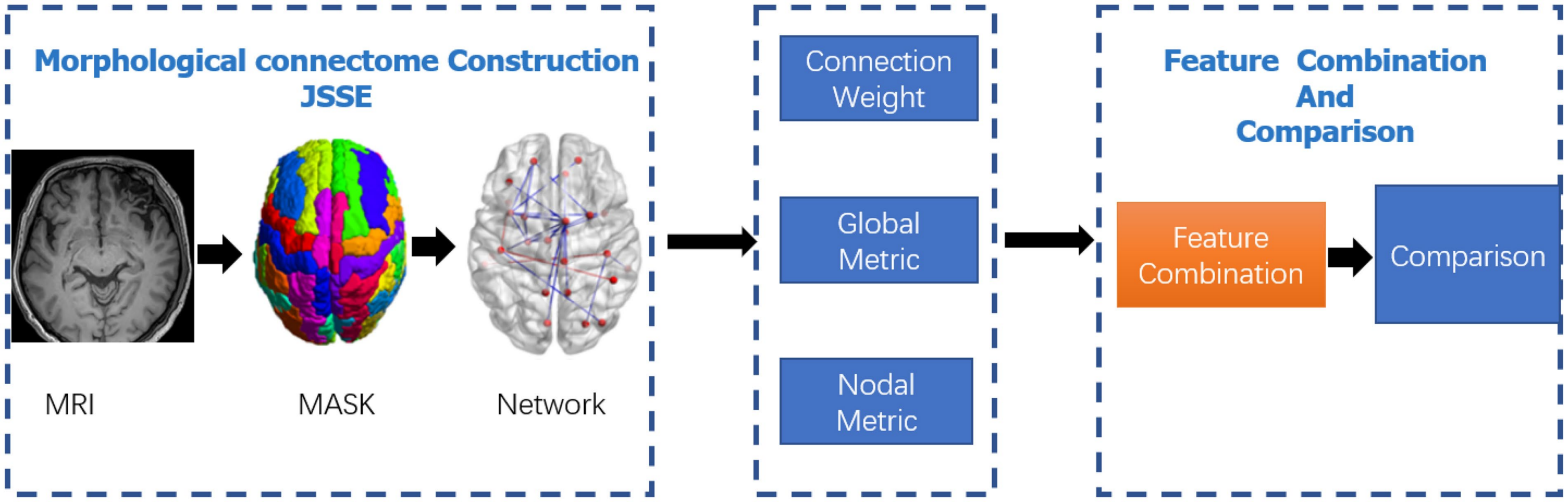
Data-processing and classification procedures adopted in the study. The JSSE was chosen to construct the morphological network. Then, the nodal and global graph metrics were computed. In the end,the MK-SVM was adopted to combine these information for comparison.

All data processing and classification procedures used in the study are shown in Figure 1.

## Results

### Demographic and clinical characteristics of the study population

The demographic and clinical characteristics of the study population are shown in Table 1. Age, education, and sex distribution were similar between the 2 groups. According to the results of the LCT, patients were divided into PD-LCT-L (UPDRS-III score improvement rate ≤ 33%) and PD-LCT-H (UPDRS-III score improvement rate > 33%).

**Table 1:** Demographic and clinical characteristics of the study population.

**Variable**	**PD-LCT-L (N = 13)**	**PD-LCT-H (N = 25)**	**p**
Age, years	63.38 ± 3.95	63.76 ± 7.85	0.873
Sex (male/female)	8/5	10/15	0.307
Education			
Illiteracy	0	1	0.095
Primary school	1	6	
Middle school	7	13	
University	5	4	
Disease duration (months)	10.23 ± 3.44	9.72 ± 3.29	0.657

Values are shown as mean ± SD or *n*.

PD-LCT-H, Parkinson disease patients with high improvement on the levodopa challenge test; LCT-L, Parkinson disease patients with low improvement on the levodopa challenge test.

### Global and local graph metrics of the brain connectome

The global graph metrics of the PD-LCT-L and PD-LCT-H groups are shown in Table 2. Compared with the PD-LCT-H group, hierarchy (***Hr***) was increased whereas assortativity, Cp, E_global_, E_local_, Lp, modularity score, synchronization, γ,λ, and σwere decreased in the PD-LCT-L group. However, the differences were not statistically significant (p>0.05).

**Table 2:** Global and local graph metrics of the brain connectome.

**Global graph metric**	**PD-LCT-L**	**PD-LCT-H**	***p***
Ar	0.1809±0.04	0.1827±0.04	0.9021
Q	17.6911±2.20	18.2621±2.45	0.4454
Hr	0.0469±0.02	0.0439±0.02	0.6520
$E_{global}$	0.2171±0.01	0.2158±0.01	0.6663
$E_{local}$	0.3621±0.01	0.3648±0.01	0.2825
$C_{p}$	0.3159±0.01	0.3196±0.01	0.2432
γ	1.0029±0.12	1.0254±0.15	0.6088
λ	0.5780±0.02	0.5805±0.04	0.7954
σ	0.7598±0.09	0.7798±0.10	0.5133
$L_{p}$	1.0962±0.07	1.1021±0.08	0.8222
$S_{r}$	−1.1078±1.65	−0.9257±1.26	0.7218

$A_{r}$, assortativity; $C_{p}$, clustering coefficient; $E_{\mathrm{global}}$, global efficiency; $E_{\mathrm{local}}$, local efficiency; Hr, hierarchy; $L_{p}$, characteristic path length; PD-LCT-H, Parkinson disease patients with high improvement on the levodopa challenge test; LCT-L, Parkinson disease patients with low improvement on the levodopa challenge test; Q, modularity score; $S_{r}$, synchronization; γ, normalized clustering coefficient; λ, normalized characteristic path length; σ, small-world.

### Consensus connections of the morphologic brain connectome

As mentioned above, we selected consensus connections with p<0.05 in each inner CV loop. As the selected connections in each loop could differ, we recorded all selected features during the entire training process—i.e., consensus connections. Specifically, we selected significant consensus connections with p<0.05 in each loop for a total of 43 (Table 3). Most of these were decreased in PD-LCT-L patients, except for those in the frontal and temporal lobe regions, which were increased. Significant consensus connections in the thalamus and putamen differed significantly between the 2 groups.

**Table 3:** Consensus connections.

		LCT-L	LCT-H	p
‘PHG.R’	‘THA.R’	1.533859	1.793755	8.47E-05
‘CUN.R’	‘THA.L’	1.610628	1.855255	0.000359
‘THA.L’	‘MTG.L’	1.538496	1.767644	0.000404
‘ORBsup.L’	‘AMYG.L’	1.621654	1.654497	0.000469
‘PCG.R’	‘HIP.L’	1.593433	1.731876	0.000654
‘CUN.R’	‘THA.R’	1.580043	1.52271	0.000672
‘IPL.R’	‘THA.R’	1.428361	1.460949	0.00072
‘DCG.L’	‘THA.R’	1.435743	1.799698	0.000749
‘THA.R’	‘MTG.L’	1.509259	1.58111	0.00116
‘CAU.L’	‘STG.L’	1.67633	1.718709	0.001267

HG.R, right parahippocampal gyrus; THA.R, right thalamus. CUN.R, right cuneus; THA.L, left thalamus; MTG.L, left middle temporal gyrus; ORBsup.L, left superior frontal gyrus; AMYG.L, left amygdala; PCG.R, right posterior cingulate gyrus; HIP.L, left hippocampus; Cun.R, right cuneus; IPL.R, inferior parietal, but supramarginal and angular gyri; DCG.L, left median cingulate and paracingulate gyri; CAU.L, left caudate nucleus; STG,L, left superior temporal gyrus.

### Degree analysis of the morphologic brain connectome

We visualized the mean degree of each node in the PD-LCT-L and PD-LCT-H groups to compare the degree distribution of the estimated brain connectomes. Specifically, there were 6 significant nodes with the average degree in the PD-LCT-L and PD-LCT-H groups (Table 4). Nodes with a standard deviation degree higher than the mean of the degree of all nodes were identified as degree hub nodes. A comparison of hub nodes between 2 groups in the same modal network revealed that most overlapped. There were also several hub nodes that corresponded to specific groups.

**Table 4:** Six significant nodes with average degree.

	**MFG.L**	**INS.L**	**AMYG.L**	**CUN.L**	**FFG.L**	**PoCG.L**
LCT-L	12.76077	12.23853	4.453462	16.89981	11.78564	17.02436
LCT-H	14.85375	14.04729	6.398854	14.89708	9.802083	14.75219
*p* value	0.043823	0.021711	0.042738	0.021208	0.019236	0.008371

AMYG.L, left amygdala; CUN.L, left cuneus; FFG.L, left fusiform gyrus; INS.L, left insula; LCT-H, high improvement on the levodopa challenge test; LCT-L, low improvement on the levodopa challenge test; MFG.L, left middle frontal gyrus; PoCG.L, left posterior cingulate gyrus.

### BC analysis of the morphologic brain connectome

To investigate the BC of the estimated morphologic brain connectome, 5 significant nodes with average betweenness in the PD-LCT-L and PD-LCT-H groups were examined (Table 5). The betweenness of left anterior cingulate cortex, left amygdala, left temporal pole of the superior temporal gyrus, and right parahippocampal gyrus tended to decrease in the PD-LCT-L group compared with the PD-LCT-H group, whereas that of the left precuneus tended to increase.

**Table 5:** Betweenness centrality.

	**ACG.L**	**PHG.R**	**AMYG.L**	**PCUN.L**	**TPOsup.L**
LCT-L	8.515045	21.04107	7.207643	64.74832	6.455661
LCT-H	13.84427	28.18066	12.3366	48.01521	12.32528
*P* value	0.012843	0.03375	0.049101	0.028623	0.044767

ACG.L, left anterior cingulate cortex; AMYG.L, left amygdala; LCT-H, high improvement on the levodopa challenge test; LCT-L, low improvement on the levodopa challenge test; PCUN.L, left precuneus; PHG.R, right parahippocampal gyrus; TPOsup.L, left temporal pole of the superior temporal gyrus.

### Ne and nodal local efficiency analysis of the morphological brain connectome

The Ne values of 4 significant nodes in the PD-LCT-L and PD-LCT-H groups are listed in Table 6. The Ne of the left middle frontal gyrus, left insula, and right thalamus showed a decreasing tendency in the PD-LCT-L group, whereas the Ne of the left precuneus showed the opposite trend. Meanwhile, NLe of the right pallidum and left thalamus tended to decrease whereas both Ne and NLe of the left fusiform gyrus increased in the PD-LCT-L group compared with the PD-LCT-R group.

**Table 6:** Nodal efficiency and nodal local efficiency.

	**Ne**				**NLe**		
	**MFG.L**	**INS.L**	**FFG.L**	**THA.R**	**FFG.L**	**PAL.R**	**THA.L**
LCT-L	0.247463	0.246564	0.243791	0.07538	0.366236	0.015702	0.141954
LCT-H	0.267427	0.265425	0.222839	0.107153	0.340273	0.043692	0.202459
P value	0.03846	0.013344	0.038538	0.027674	0.037574	0.042931	0.030709

FFG.L, left fusiform gyrus; INS.L, left insula; LCT-H, high improvement on the levodopa challenge test; LCT-L, low improvement on the levodopa challenge test; MFG.L, left middle frontal gyrus; Ne, nodal efficiency; NLe, nodal local efficiency; PAL.R, right pallidum; THA.L, left thalamus; THA.R, right thalamus.

### Classification performance

We evaluated the classification performance of the combined information and proposed JSSE method based on accuracy, sensitivity, and specificity, which were calculated with the following equations:


(7) $$Accuracy=\frac{TP+TN}{TP+FP+TN+FN}$$

(8) $$Sensitivity=\frac{Tp}{TP+FN}$$

(9) $$Specificity=\frac{TN}{TN+FP}$$

where is true positive (ie, number of positive subjects correctly classified in the identification task); FP is false positive (number of negative subjects that were incorrectly classified in the identification task); and TN and FN are the number of true negative and false negative subjects, respectively (see Table 7).

**Table 7:** Classification performance corresponding to different methods.

Method	Sen	Spe	Acc	AUC
Global(G)	72.94	100	0	0.3076
Nodal(N)	82.98	88.23	69.23	0.9343
Connection(C)	87.23	94.12	69.23	0.9547
N + G	85.11	91.17	69.23	0.9389
C + G	89.36	94.12	76.92	0.9547
C + N	93.61	100	76.92	0.9660
C + G + N	95.74	100	84.61	0.9773

SEN, sensitity; SPE, specificity

**Figure 2:**
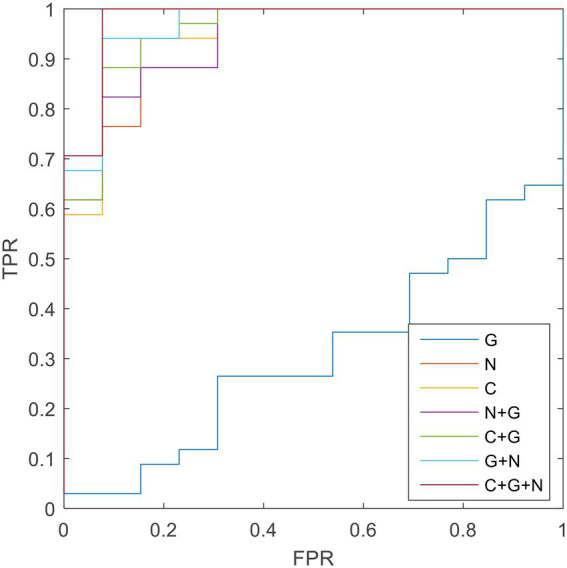
The ROC results of different methods.

To validate the combined information results, we also determined the single-kernel SVM classification based on connection and global and nodal metrics. The receiver operating characteristic curve showed that the performance of the combined information results were superior to that of the global metric (Figure 2). However, the combination of connection, global metrics, and nodal metrics did not outperform the results obtained using all 4 measurements (ie, including the LCT results).

## Discussion

The diagnosis of PD is mainly symptom-based. The heterogeneity of clinical presentation and disease course in PD reflects a complex pathogenesis and can determine the most effective treatment. Stratifying PD patients can facilitate the selection of individually tailored treatment strategies. This study investigated whether morphologic brain networks identified by MRI and analyzed by JSSE can predict the response to DBS in PD patients stratified according to improvement rates in the LCT. The results showed that while there were no significant differences in global graph metrics, the 2 groups differed with respect to DC, BC, Ne, NLe, NCp, and NLp. Thus, combining morphologic brain network characteristics and LCT results can provide detailed information regarding disease state in individual PD patients. Moreover, JSSE applied to T1-weighted MRI data can reveal inter-individual differences in brain connectivity that can inform treatment selection for patients with PD.

LCT is a valuable tool for identifying the optimal treatment for PD and is required prior to DBS. However, the test has certain limitations. First, there is no absolute standard for the results. An LR of 30% was proposed based on a placebo effect observed in one-third of patients, but a value of 33% was set in the Core Assessment Program for Intracerebral Transplantations and Core Assessment Program for Surgical Interventional Therapies in Parkinson’s Disease preoperative protocols (Defer et al., 1999); the latter value had moderately high sensitivity and a specificity of 70% for chronic levodopa, with a positive predictive value of 92.3% and negative predictive value of 32.1% (Schade et al., 2017). Second, LCT results can set certain expectations for operators, follow-up regulators, and patients (Lang and Widner, 2002). However, LCT results may be biased by the patient’s long-term oral drug use as well as psychological and other factors (Anderson and Nutt, 2011). Third, higher-than-usual doses of drug can cause gastrointestinal symptoms, but abrupt discontinuation of dopaminergic drugs can lead to neuroleptic malignant syndrome-like events in PD patients (Ikebe et al., 2003). Other factors such as oral drug dose and test time can also affect the results. For these reasons, most neurosurgeons base their assessment of the patient’s condition and the choice of treatment strategy on other modalities in addition to the LCT (Schade et al., 2017). In this study, the LCT was typically administered early in the morning when the patient was in a fasting state, and a dose of 150% of the standard morning levodopa dose was used. Improvement in motor scores compared with the “off” state was evaluated every 30 min for 4 h. Several patients complained of gastrointestinal symptoms and dizziness.

High-resolution (3.0 T or 7.0 T) MRI can provide information on pathologic changes in the brain of PD patients (Sclocco et al., 2018). Previous brain network-related studies in PD patients have mainly focused on diagnosis; analyzing the relationship between GM network topology and the GM network determined from imaging data and disease severity can provide greater resolution for early diagnosis (Suo et al., 2021b). PD patients have higher E_global_ and E_local_ than normal subjects, which are unrelated to their clinical features (Zhang et al., 2015). In studies investigating PD-associated patterns in metabolic brain networks, relatively overactive areas were considered as the source of PD brain network dysfunction (Lin et al., 2008). Some studies on the efficacy of DBS surgery found that structural and functional connectivities were independent predictors of clinical improvement (Horn et al., 2017). However, there have been no studies on the correlation between dopamine impact tests and brain networks. Our results confirm that the morphologic brain network of PD patients with different LCT test results have certain differences that warrant closer examination in future studies.

All patients enrolled in our study were diagnosed with PD by at least 1 neurologist and 1 neurosurgeon. T1-weighted MRI data were acquired before subthalamic nucleus DBS surgery. The graph theory was applied to examine individual morphologic brain networks. Both groups of patients exhibited small-world properties for global and local graph metrics of the brain connectome, and the groups did not differ in terms of nodes and global graph metrics, consistent with previous research (Jia et al., 2015). This suggests that information transmission efficiency in the whole brain was reduced in the early stage of PD disease and remained relatively stable with disease progression. We also found that the connectivity of many brain areas was weaker in the PD-LCT-L group than in the PD-LCT-H group, especially in the temporal lobe, limbic system, and thalamus, reflecting damage to these areas associated with low improvement in the LCT. Connection between the thalamus and cuneus were also altered in the PD-LCT-L group, which has been reported in patients suffering from both PD and cognitive impairment (Li et al., 2020). A correlation has been observed between atrophy of thalamic neurons, reduced thalamic volume, and cognitive function (Chen F. X. et al., 2016). As a key hub of the default mode network, the precuneus is involved in many advanced cognitive functions; impaired connections in the precuneus reflected a decline in the cognitive level of patients in the PD-LCT-L group. Among indicators of complex network operation, the cluster coefficient measures the degree of collectivization of the network, node degree describes centrality in the network, and E_global_ and E_local_ represent the network’s global and local transmission capacities, respectively (He and Evans, 2010).

**Figure 3:**
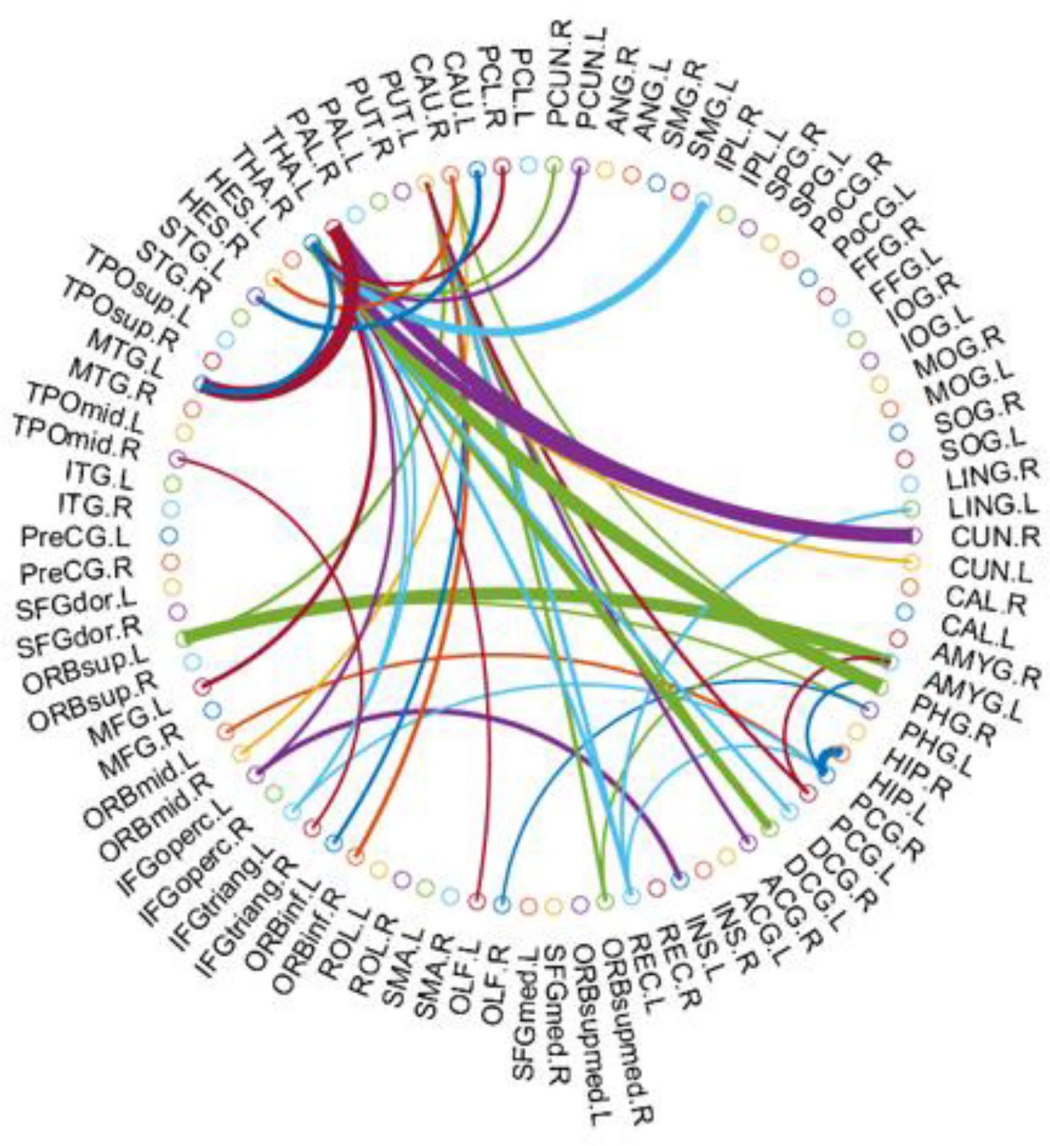
The most consensus connections mapped on the International Consortium for Brain Mapping (ICBM) 152 template using the BrainNet Viewer software package http://nitrc.org/projects/bnv/ and circularGraph, shared by Paul Kassebaumb http://www.mathworks.com/matlabcentral/fileexchange/48576-circulargraph. The connectivity matrices of the fully connected network of PD-LCT -L compared to PD-LCT-H are shown. The 43 most significant connections were retained.

Compared with the PD-LCT-H group, the PD-LCT-L group showed increased connectivity in a few areas of the frontal and temporal lobes, and the cluster coefficients of the dorsolateral superior frontal gyrus and DC, Ne, and NLe of the left fusiform gyrus were increased; moreover, the DC of the left cuneus and left postcentral gyrus and BC of the precuneus were also increased. These brain regions are all related to cognition and movement.From the Figure 3, we observed that the brain areas with increased connectivity in the PD-LCT-L group tended to be on the left side rather than on the right, and the left fusiform gyrus connectome showed a compensatory increase in connectivity. In terms of consensus connections, the right parahippocampal gyrus–right thalamus network was the most prominent. As the main cortical input to the hippocampus, this pathway plays an important role in cognition and emotion, which explains the anxiety and depression observed in patients with poor drug control (Zhang et al., 2019). Another important pathway for consensus connections identified in our study was the orbitofrontal cortex–amygdala and Heschl’s gyrus. The orbitofrontal cortex is essential for processing visual, spatial, and emotional information (Rolls, 2019). We found that this brain area was closely linked to the parietal occipital lobe and was also a central node in the morphologic brain network. Heschl’s gyrus is located in the primary auditory cortex, occupying Brodmann areas 41 and 42; it is the first cortical structure to process incoming auditory information (Abdul-Kareem and Sluming, 2008). We observed significant differences in mood, anxiety, and depression between PD-LCT-L and PD-LCT-H patients; the connection networks related to motor disorders validated in our study may provide insight into the pathophysiology of certain emotional disorders and their relationship to clinical symptoms in PD. We also found that the connection between the thalamus and putamen differed between the 2 groups, with fewer connections in the PD-LCT-L group; this was previously shown to be related to the degree of cognitive dysfunction and tremor severity in some patients with PD (Halliday, 2009; Li et al., 2021b, 2022). Taken together, these findings provide an anatomic basis for evaluating the clinical symptoms of PD as well as potential imaging biomarkers for diagnosis.

There were several limitation to this study.We havenot adopt multiple comparison correction analysis, such as Bonferroni correction, false discovery rate correction, and we will promote in the future work. Additionally, the data were analyzed retrospectively and could not be stratified according to different PD clinical symptoms. A control cohort would be explored in future investigations.

## Conclusion

The results of this study show that JSSE based on MRI data can be used in conjunction with LCT results to identify candidates for DBS among patients with PD. Our findings also provide new insight into abnormalities in the morphologic brain network in PD that can inform individualized treatment decisions.

## Data availability statement

The original contributions presented in the study are included in the article/supplementary material, further inquiries can be directed to the corresponding authors.

## Ethics statement

The studies involving human participants were reviewed and approved by the Institutional Review Board of Huashan Hospital. The patients/participants provided their written informed consent to participate in this study.

Written informed consent was obtained from the individual(s) for the publication of any potentially identifiable images or data included in this article.

## Author contributions

All authors contributed to study conception and design. YX, CG, and BW were performed the material preparation and data collection and analysis and drafted the manuscript. JW and LL commented on previous versions of the manuscript. All authors read and approved the final manuscript.

## Funding

This study was sponsored by the Shanghai Municipal Science and Technology Major Project (no. 2018ZDZX01); National Natural Science Foundation of China (no. 82001202 to BW); and Shanghai Municipal Commission of Health and Family Planning Science and Research Subjects (no. 202140464).

## Conflict of interest

The authors declare that the research was conducted in the absence of any commercial or financial relationships that could be construed as a potential conflict of interest.

## Publisher’s note

All claims expressed in this article are solely those of the authors and do not necessarily represent those of their affiliated organizations, or those of the publisher, the editors and the reviewers. Any product that may be evaluated in this article, or claim that may be made by its manufacturer, is not guaranteed or endorsed by the publisher.

## Abbreviations

AAL: automated anatomical labeling
BC: betweenness centrality
Cp: clustering coefficient
DBS: deep brain stimulation
DC: degree centrality
E*_global_*: global efficiency
E*_local_*: local efficiency
FN: false negative
FP: false positive
JSSE: Jensen–Shannon divergence similarity estimation
KL: Kullback–Leibler
LCT: levodopa challenge test
Lp: characteristic path length
MK-SVM: multiple kernel support vector machine
MRI: magnetic resonance imaging
PD: Parkinson disease
PDF: probability density function
PET: positron emission tomography
RHKS: reproducing Hilbert kernel space
ROI: region of interest
TN: true negative
TP: true positive
UPDRS: United Parkinson’s Disease Rating Scale
γ: normalized clustering coefficient
λ: normalized characteristic path length
σ: small-world
